# La tumeur de Buschke-Löwenstein

**DOI:** 10.11604/pamj.2020.36.359.13292

**Published:** 2020-08-28

**Authors:** Youssef Kadouri, Yassine Nouini

**Affiliations:** 1 Université Mohamed V, Faculté de Médecine et de Pharmacie de Rabat Maroc, Hôpital Ibn Sina, Service d´Urologie A, Rabat, Maroc

**Keywords:** Tumeur, Buschke, Löwenstein, Tumor, Buschke, Löwenstein

## Abstract

Buschke-Lowenstein’ tumor (BLT) or giant condylomata acuminata (GCA) is a pseudo-epithelial proliferation belonging to the verrucous carcinomas. It was first described in 1896. In 1925 Buschke and Lowenstein showed that it was characterized by disease-specific features. It is caused by human papilloma virus (HPV) a sexually transmitted infection mainly affecting the anogenital region. It can result in progressive degeneration and recur after treatment. Buschke-Lowenstein’s tumor is relatively rare and it is always preceded by condylomata acuminata. Its annual incidence appears to be 0.1% among the sexually active adult population. It can occur at any age after puberty but mainly between the 4^th^and 6^th^decades. The infection can affect both sexes but it most commonly occurs in males. Development, persistence, and recurrence rates of condylomas largely depend on the host’s immune status. Immunodepression, chronic inflammation, lack of hygiene and HIV infection appear to be risk factors for this disease. BLT is known to be caused by papillomavirus and, in particular, by HPV serotypes 6, 11. Prevention is essential and it should be based on the treatment of condylomata acuminata and the battle against sexually transmitted diseases. Early wide excision is mandatory. We here report the case of a 55-year old patient with a history of sexual vagrancy and recurrent urethritis, presenting with penile tumor progressing over the last 8 years. Clinical examination showed several fetid, painless, invasive, cauliflower papillomatous tumor lesions involving the root and ventral face of the penis. Lymph nodes were free. Serological tests for HIV, syphilis and hepatitis B and C were negative. Treatment was based on surgical excision with skin coverage using adjacent skin. Histological examination of the surgical specimen showed acute papillomatous hyperplasia of the epidermis and some koilocytes suggesting giant condyloma. At 2-year follow-up the patient didn’t show any recurrence.

## Image en médecine

La tumeur de Buschke-Löwenstein (TBL) ou condylome acuminé géant (CAG) est une prolifération pseudo-épithéliomateuse appartenant au groupe des carcinomes verruqueux. Sa première description remonte à 1896. C´est en 1925 que Buschke et Löwenstein en ont fait une entité caractérisée. Elle est d´origine virale (HPV), de transmission sexuelle atteignant surtout les zones ano-génitales. Elle est caractérisée par son potentiel dégénératif et son caractère récidivant après traitement. La TBL est une affection relativement rare et toujours précédée de condylomes acuminés, son incidence annuelle semble être de 0,1% parmi la population adulte active sexuellement. Elle survient à tout âge après la puberté et prédomine entre les 4^e^ et 6^e^ décennies. L´infection peut atteindre les deux sexes, elle se voit fréquemment chez le sexe masculin. Le développement, la persistance et les récidives des condylomes dépendent largement du statut immunitaire de l´hôte. L´immunodépression, l´inflammation chronique, le manque d´hygiène et l´infection à VIH semblent être des facteurs de risque de cette affection. L´implication du papillomavirus et en particulier de ses sérotypes l´HPV 6 et 11 est admise dans la genèse de la TBL. Sa prévention est impérative, basée sur le traitement des condylomes acuminés et la lutte contre les maladies sexuellement transmissibles. Le traitement doit être précoce, il est essentiellement chirurgical nécessitant une large exérèse. Nous rapportons le cas d´un patient âgé de 55 ans, ayant comme antécédent une notion de vagabondage sexuel et des urétrites à répétition, qui consulte pour une tumeur pénienne évoluant depuis 8 ans. L´examen clinique notait la présence plusieurs lésions tumorales infiltrées, papillomateuses en chou-fleur intéressant la racine et la face ventrale de la verge, fétides et indolores. Les aires ganglionnaires étaient libres. Les sérologies VIH, syphilitique et des hépatites B et C étaient négatives. Le traitement a consisté en une exérèse chirurgicale avec un recouvrement cutané par la peau adjacente. L´examen histologique de la pièce d´exérèse a retrouvé une importante hyperplasie papillomateuse de l´épiderme et quelques koïlocytes en faveur d´un condylome géant. Après 2 ans de recul nous n´avons pas constaté de récidive.

**Figure 1:**
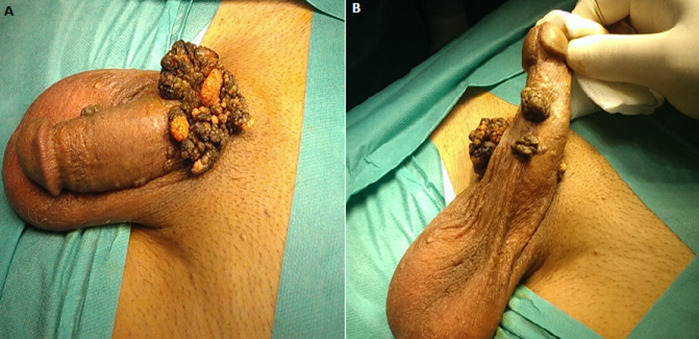
A) tumeur de Buschke - Lowenstein: lésion en chou-fleur krato-verruqueuse au niveau de la racine de la verge; B) lésions en chou-fleur krato-verruqueuses au niveau de la face ventrale de la verge

